# Traditional trapping methods outperform eDNA sampling for introduced semi-aquatic snakes

**DOI:** 10.1371/journal.pone.0219244

**Published:** 2019-07-02

**Authors:** Jonathan P. Rose, Cara Wademan, Suzanne Weir, John S. Wood, Brian D. Todd

**Affiliations:** 1 Department of Wildlife, Fish & Conservation Biology, University of California, Davis, Davis, California, United States of America; 2 Department of Medicine and Epidemiology, University of California, Davis, Davis, California, United States of America; 3 Pisces Molecular, LLC, Boulder, Colorado, United States of America; Universitat Zurich, SWITZERLAND

## Abstract

Given limited resources for managing invasive species, traditional survey methods may not be feasible to implement at a regional scale. Environmental DNA (eDNA) sampling has proven to be an effective method for detecting some invasive species, but comparisons between the detection probability of eDNA and traditional survey methods using modern occupancy modeling methods are rare. We developed a qPCR assay to detect two species of watersnake (*Nerodia fasciata* and *Nerodia sipedon*) introduced to California, USA, and we compared the efficacy of eDNA and aquatic trapping. We tested 3–9 water samples each from 30 sites near the known range of *N*. *fasciata*, and 61 sites near the known range of *N*. *sipedon*. We also deployed aquatic funnel traps at a subset of sites for each species. We detected *N*. *fasciata* eDNA in three of nine water samples from just one site, but captured *N*. *fasciata* in traps at three of ten sites. We detected *N*. *sipedon* eDNA in five of six water samples from one site, which was also the only site of nine at which this species was captured in traps. Traditional trapping surveys had a higher probability of detecting watersnakes than eDNA surveys, and both survey methods had higher detection probability for *N*. *sipedon* than *N*. *fasciata*. Occupancy models that integrated both trapping and eDNA surveys estimated that 5 sites (95% Credible Interval: 4–10) of 91 were occupied by watersnakes (both species combined), although snakes were only detected at four sites (three for *N*. *fasciata*, one for *N*. *sipedon*). Our study shows that despite the many successes of eDNA surveys, traditional sampling methods can have higher detection probability for some species. We recommend those tasked with managing species invasions explicitly compare eDNA and traditional survey methods in an occupancy framework to inform their choice of the best method for detecting nascent populations.

## Introduction

Determining the distribution of an introduced species is an important step for planning control and eradication efforts. For a widely distributed introduced species, effort might be focused on limiting its further spread. For an introduced species currently restricted to a small area in its non-native range, effort might be expended to eradicate the nascent population. Given limited resources, a survey method that has a high probability of detecting the introduced species while requiring the least effort is highly desirable. In the past decade, many studies have begun using environmental DNA (eDNA) to survey for the presence of secretive, endangered, or invasive species [1–3]. Although eDNA can be more effective at documenting the presence of aquatic species than traditional survey methods [1,4], many studies have not accounted for the imperfect detection of eDNA assays using modern statistical methods ([5,6] but see [7,8]).

While eDNA sampling has come to prominence, ecology has also benefited from the development of site-occupancy models that account for false-negatives in wildlife surveys [9]. In a standard occupancy study, repeated surveys are conducted for some or all sites, producing a sequence of detections and non-detections at each site. Analyzing the results of detection/non-detection surveys in an occupancy framework separates the detection process (detected or not detected) from the occupancy process (present or absent) and provides an estimate of the certainty that a site is unoccupied, given that the species of interest is not detected. Estimating the probability that a site is occupied is especially valuable for the conservation of imperiled species or for the management of invasive species [10–12]. Incorrectly concluding that a species is absent from a site could have undesirable consequences; habitat may be developed when it is occupied and needed by an endangered species, or it may be deemed unoccupied by a non-native species that left undisturbed could continue to increase in abundance and disperse.

Occupancy methods have been widely adopted by ecologists and wildlife biologists, with new methods to handle different types of data and sources of error that are published regularly [13,14]. Presence-absence surveys using eDNA are equally well-suited to occupancy modeling as are traditional visual, auditory, and trapping methods. Collecting replicate water samples from a site does not greatly increase the survey effort expended, but the additional information gained by modeling the detection process can be valuable. Recent studies have shown that analyzing replicate eDNA samples and PCR amplifications in an occupancy framework provides information about detection probability—at multiple levels of sampling—and the amount of replication necessary to have confidence in ruling out false absences [7,8,15]. Combining eDNA with traditional survey methods like traps or visual searches may have additional benefits. For example, combining two survey methods can enable the use of occupancy models that account for false positives as well as false negatives [16]. Thus, eDNA sampling might be better viewed as a complement to, rather than a replacement for, traditional survey methods.

In this study, we compare eDNA sampling to trapping as a means of detecting the presence of two non-native, semi-aquatic watersnake species (*Nerodia fasciata* and *Nerodia sipedon*) in freshwater habitat in the Central Valley of California. Our goal was to determine whether eDNA sampling could be as reliable for documenting the presence of these non-native species as is trapping with aquatic funnel traps. We analyze data from repeated surveys in an integrated occupancy model to estimate the detection probability of each method and the occupancy probability at our study sites. Our method allows both survey types to inform the true occupancy status of a site, providing greater certainty that a site is unoccupied if the species goes undetected. Our results provide valuable information about the distribution of these two non-native snake species in California, as well as an example for how eDNA and traditional sampling methods can be combined to improve inference about species distributions.

## Methods

### eDNA sample collection and processing

**Fig 1:**
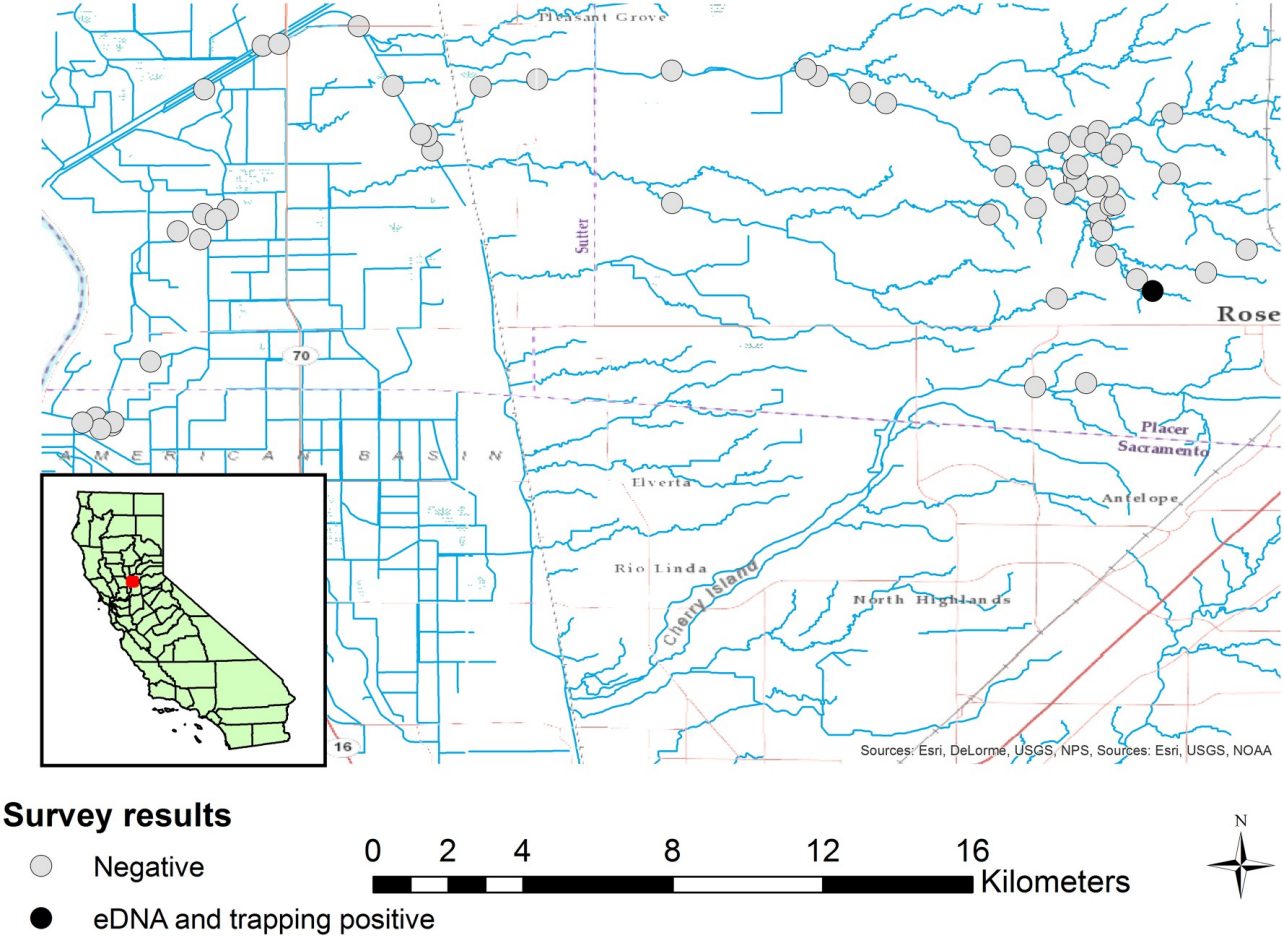
Location of sample sites for *Nerodia sipedon*. 61 sites sampled for the presence of *Nerodia sipedon* using eDNA, and for a subset of nine sites, aquatic funnel traps.

**Fig 2:**
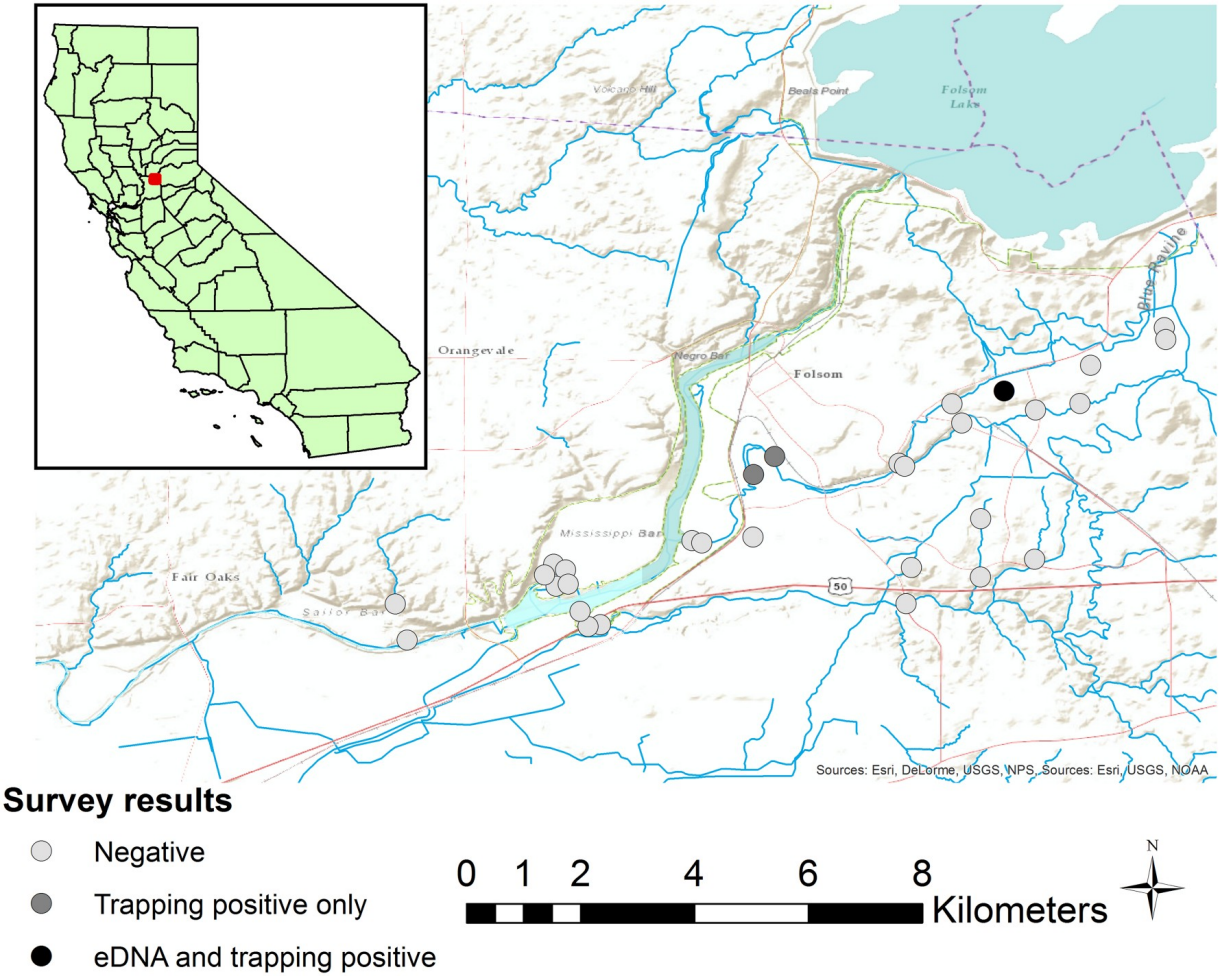
Location of sample sites for *Nerodia fasciata*. 30 sites sampled for the presence of *Nerodia fasciata* using eDNA, and for a subset of 10 sites, aquatic funnel traps.

We used satellite imagery and field visits to identify wetland habitats, including pools in intermittent streams, ponds, lakes, and flowing streams that appeared suitable for *Nerodia* and were within approximately 10 km and 20 km of known populations of *N*. *fasciata* and *N*. *sipedon* respectively. Our final sample locations were not chosen randomly; rather we chose sites based on the presence of water in spring and summer 2015 (at the end of an extreme drought, see [17]) and whether we could obtain permission to access the wetland habitat. *Nerodia* are active and foraging in water bodies from mid-March through September in California, and therefore eDNA would be expected to be available at the time of sampling if a site was occupied. We collected water samples from a total of 91 sites in Placer, Sacramento, and Sutter counties, California, USA, from April through August 2015. These 91 sites included two sites where *N*. *sipedon* was previously known to occur in Roseville, California, USA, [18,19] and 59 sites of unknown status near these previously occupied sites and along a potential dispersal corridor into sensitive habitat for native wildlife (Fig 1). Also included were two sites where *N*. *fasciata* was known to occur in Folsom, California, USA and 28 sites of unknown status near these occupied sites (Fig 2). Most sites (77) were lentic (ponds, lakes, or still pools in ephemeral streams), but 7 sites near the known range of *N*. *sipedon* and 7 sites near the known range of *N*. *fasciata* were flowing streams.

At each site, we collected between three and nine 500 mL water samples by submerging a 1 L Nalgene^™^ plastic bottle in water. Replicate water samples from the same site were collected within 100 m of one another, with the distance between samples depending on the size of the water body. For 70 sites, we collected all water samples during a single visit, but samples were collected from the remaining 21 sites on two separate occasions. Of 21 sites visited multiple times, 15 had visits separated by eight or fewer days, two sites were visited approximately two weeks apart, whereas at the remaining four sites, the repeat visits occurred 35 days apart. We assumed that the time interval between repeat visits for these 21 sites was short enough that no sites changed occupancy status between visits and the closure assumption of our single-season occupancy model was not violated [9]. We also collected and analyzed six field-negative water samples, which consisted of 500 mL of distilled water that was poured into a 1 L bottle and transported in a cooler alongside field-collected water samples. These field-negative water samples were then processed following the same protocol as the rest of the field-collected samples as a test for cross-contamination between water samples.

We vacuum filtered all water samples through 250 mL Nalgene^™^ Analytical Test Filter Funnels with 0.45 μm cellulose nitrate filters. For less than 10% of water samples, we could not filter all 500 mL because sediment or organic matter clogged the filter. After filtration, we stored filters in 2 mL microcentrifuge tubes in a -20 °C freezer until DNA extraction. We extracted DNA from the filters using a Qiagen DNeasy Blood & Tissue Kit (Qiagen N.V., Venlo, Netherlands). After extraction, we treated samples with Gene Releaser (BioVentures, Inc, Murfreesboro, TN, USA) to remove PCR inhibitors because many water samples were from stagnant bodies of water with suspended sediment [20]. All eDNA samples were processed in a clean lab with lab benches sterilized daily using 10% bleach and covered with fresh bench paper. All equipment that touched water samples (e.g., Nalgene bottles, forceps used to manipulate filters) was cleaned with 50% bleach and rinsed three times with distilled water between uses, following standard protocols [21]. Equipment used during the filtering process that did not directly contact samples but was downstream of the sample (e.g., vacuum filtering flasks, rubber stoppers, vacuum tubing) was washed with 10% bleach and rinsed three times with distilled water between samples. New nitrile gloves were used to process each sample.

### eDNA qPCR assay

**Fig 3:**
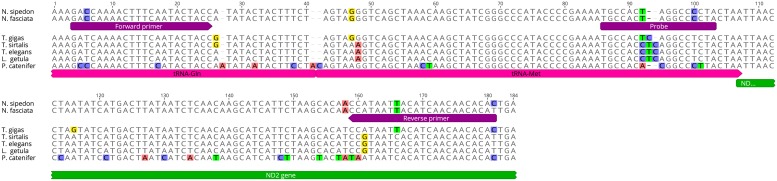
Sequence alignment of *Nerodia* and non-target species with qPCR primer and probe locations.

The close taxonomic relationship between the two target, non-native *Nerodia* species and native *Thamnophis* species, also within the sub-family Natricinae, made finding sites for qPCR primers and probes that could distinguish these two genera difficult. Using alignments of the limited available mitochondrial sequence data for these species from GenBank and BOLD, supplemented with additional, *de novo* mitochondrial sequence data we obtained for the control region, ND2 and CO1 regions from these species, as well as two less closely related snake species, *Pituophis catenifer* and *Lampropeltis getula* (data not shown), we identified a 173 bp sequence across tRNA-GLN, tRNA-MET and the 5’ end of the ND2 gene that was shared between *N*. *fasciata* and *N*. *sipedon*, but not *Thamnophis elegans*, *T*. *gigas*, or *T*. *sirtalis* (Fig 3). We developed primers and an internal hydrolysis probe for this target sequence using Geneious software version 6.1.8 (https://www.geneious.com) (Table 1) and tested the specificity of the primers *in silico* by performing a BLAST search on GenBank; there was no significant homology to any non-target species other than *Thamnophis*. Both primers used blocked, RNase-H cleavable 3’ moieties, to increase the specificity of the assay [22]. The internal hydrolysis probe was labeled at the 5’ end with reporter dye FAM (6-carboxyfluorescein), and at the 3’ end with quencher dye BHQ-1 plus (LGC Biosearch Technologies, Petaluma, CA, USA). We tested the specificity of the assay using DNA extracted from tissue samples of target species *N*. *fasciata* (University of California, Davis Museum of Wildlife and Fish Biology [MWFB] Catalogue numbers: F018, F024) and *N*. *sipedon* (MWFB Catalogue numbers: JMM20, JMM21), and non-target native species *T*. *elegans* (University of California, Berkeley Museum of Vertebrate Zoology [MVZ] Catalogue numbers: MVZ 249125, MVZ 250252, MVZ 253485), *T*. *gigas* (U.S. Geological Survey tissue samples; U.S. Fish and Wildlife Service Permit TE-157216-2), *T*. *sirtalis* (MWFB Catalogue numbers: JMM3), *Pituophis catenifer* (MWFB Catalogue numbers: ATH1027, JMM4), and *Lampropeltis getula* (MVZ Catalogue numbers: MVZ 170806, MVZ 175859, MVZ 232673).

**Table 1:** Forward and reverse primers and probe sequences used in RNase-H dependent qPCR assay for detecting *N*. *fasciata* and *N*. *sipedon*.

Oligonucleotide	Sequence
Forward rh Primer	5' GACCAAAACTTTCAATACTACCrATATAA/C3sp/
Reverse rh Primer	5' GTGTTGTTGATGTAATTATGGrUTGTG/C3sp/
Probe	5' [FAM]-TAGGGGCCTAGTGGCA-[BHQ-1 plus]

rA and rU indicate an RNA base instead of DNA, and C3sp represents a 3′ C3 spacer.

We validated the sensitivity and efficiency of the assay by using 10-fold serial dilutions of a positive control plasmid. The control plasmid was pUC57-Kan containing a synthetic insert of the 173 bp *Nerodia* sequence, flanked by an additional 10 bp of *Nerodia* sequence on either end to minimize the effect of differences between the adjacent plasmid and *Nerodia* sequences. The control plasmid was linearized using restriction digestion at a DraI site in the plasmid backbone distal to the insert. We made 10-fold serial dilutions of the linearized plasmid from 1.3 x 10^6^ molecules/uL to 1.3 molecules/uL. We determined the exact concentration of the most dilute standard using a Poisson P0 experiment: we ran 88 replicate qPCR reactions of a 1:2 dilution of the most dilute standard and counted the total number of replicates that failed to amplify. We then used the failure rate (0.273, or 24/88 replicates) to calculate the average rate of success (1.3 molecules/uL). We then plotted the Cq value against the log of the dilution to create a standard curve. Using traditional calculation methods, we were able to obtain the sensitivity and efficiency from the y-intercept and slope, respectively, of the resulting curve. The reaction efficiency, based on plotting Cq values for the dilution series, was between 90% and 105% with r^2^ values > 0.98. The sensitivity of the assay was approximately 3 molecules/reaction. Preliminary analysis of two types of positive control samples (buffer containing plasmids with the target mtDNA sequence and water from aquaria containing *N*. *sipedon*) using the RNase-H dependent qPCR assay indicated the presence of watersnake DNA at or below the assay’s limit of detection and demonstrated the need for pre-amplification (Table 2). To address this, eDNA from all field samples was pre-amplified using conventional PCR prior to qPCR to increase the concentration of watersnake DNA, if present [23]. After pre-amplification, we ran all samples with the RNase-H dependent qPCR assay, which is designed for high specificity to *Nerodia* mtDNA so that mtDNA from closely related native species (e.g., *Thamnophis* species) would not be falsely detected. Assaying for eukaryotic 18S (Hs99999901_s1, Thermo Fisher Scientific, Waltham, MA, USA) served as a DNA extraction control, ensuring efficient DNA extraction if detection was within one standard deviation from the average of all samples. Samples with 18S Cq values falling outside of this range could be considered failing quality control. We tested for PCR inhibition by including an internal positive control (TaqMan Exogenous Internal Positive Control, 4308323, Applied Biosystems, Foster City, CA, USA) in each qPCR reaction. No cutoff values were established for the internal positive control, but detection had to be present to include any sample in the study. We ran our qPCR assay for 50 cycles and considered a sample positive for *Nerodia* eDNA if amplification crossed the fluorescence threshold within 35 PCR cycles. We used positive (diluted plasmid) and negative (Diethylpyrocarbonate-treated water) qPCR and preamplification controls on every plate containing samples. Negative and positive qPCR controls performed as expected; there was no evidence of primer/dimers or non-specific binding of primers or probe, and diluted plasmid containing the target sequence successfully amplified.

**Table 2:** Comparison of single-step qPCR assay to two-step pre-amplification with conventional PCR followed by qPCR for detection of watersnake eDNA.

Sample Type	Cq value	
	qPCR only	Pre-amp + qPCR
Watersnake plasmid	41.97	17.63
Lab-Positive 1	-	15.85
Lab-Positive 2	-	17.94
Lab-Positive 3	-	20.20

Watersnake plasmid was the target sequence inserted into NS4 plasmid at a concentration of 1.3 x 10^3^ molecules/uL.

A “-”indicates no amplification of target DNA.

### Pre-amplification

Each conventional PCR reaction contained 5 μl of extracted DNA, Advantage 2 SA PCR buffer, Advantage 2 Polymerase mix (639201, Clontech, Mountain View, CA, USA), 0.2 mM each dNTP (10297–018, Invitrogen, Waltham, MA, USA), 10 μM each non-rh primer (Eurofins MWG Operon, Louisville, KY, USA, primers minus the rh spacer and substituting DNA bases for RNA bases, Table 1), and eukaryotic 18S for a total volume of 50 μl. Preamplification was performed on a SimpliAmp Thermal Cycler (Applied Biosystems, Foster City, CA, USA). Standard amplification conditions were as follows: 1 min at 94°C, 25 cycles of 15 s at 94°C, 15 s at 51°C, and 45 s at 70°C, then 5 min at 70°C, and 10 min at 4°C.

### qPCR reaction

Each qPCR reaction contained 5 μl of pre-amplified DNA (diluted 1:10 with diethylpyrocarbonate-treated water), Core Buffer, 3 mM Mg, 0.35 pmoles ROX reference Dye, 2 U TaqGold (N8080247, Applied Biosystems), 16 nmoles dNTPs (N0447L, New England BioLabs, Ipswich, MA, USA), 10 pmoles forward and reverse primers (PM607 and PM585, Integrated DNA Technologies, Skokie, IL, USA), 10 pmoles probe, 5.2 mU RNase H2 enzyme (11-02-12-01, Integrated DNA Technologies, Skokie, IL, USA), Exogenous IPC Mix, and Exogenous IPC DNA for a total volume of 20 μl. qPCR was performed on an automated fluorometer (ABI PRISM 7900 HTA FAST, Thermo Fisher Scientific). The following amplification conditions were used: 9 min at 95°C, 50 cycles of 30 s at 95°C and 1 min at 63°C. Fluorescent signals were collected during the annealing phase and Cq values extracted at adjusted baselines and thresholds appropriate for FAM (watersnake assay) and VIC (internal positive control) fluorophores.

### eDNA analysis

We tested nine water samples from six sites, six water samples from one site, and three samples from each of the remaining 84 sites, for a total of 312 field samples. We chose to run all nine samples from two sites previously known to be occupied by *N*. *fasciata* and four sites nearby, because more replicates from occupied sites should improve our estimates of detection probability using eDNA [24,25]. We ran an initial test of the assay on 102 eDNA samples (93 field samples from 31 sites, 2 field-negative samples, 2 lab-negative samples, and 5 lab-positive samples), with each sample run in duplicate. Each duplicate qPCR run produced the same result, and all controls produced the expected result. Thereafter we ran a single qPCR assay for each water sample because we believed it was a better use of our limited budget to analyze replicate water samples than replicate qPCR runs [7].

To test the efficacy of the combined pre-amplification and qPCR assay, we also analyzed a small sample of lab-positive (*N*. *sipedon*) and lab-negative (*T*. *elegans* and *T*. *sirtalis*) controls. For both controls, we confined snakes to small aquaria filled with 2 L of distilled water for 6 hours, after which we removed the snakes and processed 500 mL water samples from the aquaria following same protocol as field water samples. We used one individual each of *T*. *elegans* and *T*. *sirtalis* as negative controls because both species occur in wetland habitats in our study region and are closely related to *Nerodia*. The negative controls were necessary to establish that our qPCR assay would not produce false positives by amplifying *Thamnophis* eDNA. We filtered, extracted, and analyzed two 500 mL lab negative water samples each for *T*. *elegans* and *T*. *sirtalis* and five total lab-positive water samples from three *N*. *sipedon* (two samples each for two individuals, and one sample from the third).

### Aquatic trapping surveys

As a second method of detecting the presence of *Nerodia* in suitable wetland habitat, we trapped a subset of ten eDNA sample sites for *N*. *fasciata* and nine eDNA sample sites for *N*. *sipedon* during July and August 2015. At each site, we deployed 30 plastic aquatic funnel traps (Model 700 Minnow Trap, Gator Buckets, New Market, IN, USA) for five nights, resulting in 150 trap-nights of sampling effort, following an earlier occupancy study of *N*. *fasciata* in its native range [26]. Aquatic funnel traps have proven effective at sampling both *Nerodia* species in their native and introduced ranges [18,26,27]. All trapping took place after water samples had been collected from a site, to prevent introduction of *Nerodia* DNA that could contaminate our eDNA samples.

### Occupancy modeling

We fit an integrated single-season occupancy model to our field data to estimate detection probability (*p*) and the occupancy rate (ψ) of our surveyed sites. We modified code in [28] to enable both types of detection/non-detection data (eDNA and trapping) to inform the true occupancy state (*z*) of a site (S1 File). The detection/non-detection data are a series of binary values for each site with a one representing that watersnake DNA was detected in a single eDNA sample or that a watersnake was captured during one trap-night of sampling and a zero representing a negative eDNA sample or a trap-night during which no watersnakes were captured. The model included separate parameters for the detection probability of eDNA samples (*p*_*eDNA*_) and the detection probability of sampling with aquatic funnel traps (*p*_*trap*_). We compared *p*_*eDNA*_ and *p*_*trap*_ by calculating the posterior probability that the difference between the two detection probabilities (Δ*p*) was greater than zero. We also included an effect of species on the eDNA and trapping detection probabilities to test whether there was any difference in the detection probability between species for either method. We calculated the probability of detecting a watersnake at least once in *n* samples, given that a site was occupied, as p* = 1 − (1 − *p*)^*n*^. We also fit separate occupancy models to the eDNA data or the trapping data alone, for comparison to the model that integrated both data sources.

We fit models in JAGS [29] using the “runjags” package [30] in R version 3.5.1 [31]. We used uninformative priors for mean detection probabilities and weakly regularizing priors for species effects on detection probabilities (Table 3). All code and data are available in S1 File. We ran models on four chains for 25,000 sampling iterations, after discarding a burn-in of 10,000 iterations. We thinned the resulting chains by 1/10^th^, resulting in 10,000 samples from the posterior distribution. We evaluated the mixing of chains by inspecting trace plots; all chains appeared well-mixed. We tested for convergence using the Brooks-Gelman $\hat{R}$ statistic [32] and the effective sample size; all parameters had an $\hat{R}$ value < 1.01 and an effective sample size > 9000. For all parameters, we present posterior medians followed by symmetrical 95% credible intervals (CRI) in parentheses.

**Table 3:** Parameters, prior distributions, and posterior summaries for detection and occupancy parameters from a joint occupancy model that integrates both trapping and eDNA survey results.

Parameter	Description	Prior	Median	SD	2.50%	97.50%
*p*_eDNA_	Mean detection probability of eDNA	Uniform(0,1)	0.44	0.16	0.15	0.75
*p*_trap_	Mean detection probability of trapping	Uniform(0,1)	0.74	0.13	0.48	0.95
α_eDNA_	Intercept of eDNA linear predictor	--	-0.24	0.73	-1.72	1.15
α_trap_	Intercept of trapping linear predictor	--	1.04	0.70	-0.28	2.45
β_fas, eDNA_	Species effect on *p*_eDNA_	Normal(0,1)	-1.55	0.73	-2.92	-0.08
β_fas, trap_	Species effect on *p*_trap_	Normal(0,1)	-0.92	0.72	-2.35	0.47
ψ	Occupancy probability	Uniform(0,1)	0.07	0.04	0.02	0.15
N_all_	Estimated number of sites occupied by watersnakes	--	5	2.2	4	10
N_fas_	Estimated number of sites occupied by *N*. *fasciata*	--	4	1.17	3	6
N_sip_	Estimated number of sites occupied by *N*. *sipedon*	--	1	1.54	1	5

### Animal Care

Snakes captured as part of the aquatic funnel trapping study were released at their site of capture the same day. Snakes used as part of lab eDNA control experiments were kept in captivity for up to 48 hours. After lab eDNA control experiments were finished, *T*. *elegans* and *T*. *sirtalis* individuals were released at their site of capture. The three *Nerodia sipedon* used for lab-positive controls were humanely euthanized (at the request of the California Department of Fish and Wildlife as part of ongoing *Nerodia* eradication efforts) using an overdose of isoflurane, as approved by the UC Davis Institutional Animal Care and Use Committee (IACUC). All animals were handled in accordance UC Davis IACUC protocol #17801.

## Results

All five lab-positive control water samples tested positive for *Nerodia* eDNA in our qPCR assay. None of the four lab-negative control water samples from aquaria containing native *Thamnophis* returned positive results in our qPCR assay, showing the assay did not amplify DNA from co-occurring native species closely related to *Nerodia*. Also, all eight field-negative water samples tested negative for *Nerodia* eDNA, indicating that cross-contamination of samples transported together and processed on the same equipment was not an issue.

Neither species of *Nerodia* was widely distributed beyond a few localized populations, based on the results of both eDNA and trapping surveys. We captured *N*. *sipedon* at one of nine sites that were trapped, and this was the only site out of 61 that returned positive results in our eDNA assay as well (Fig 1). This site (WOHS) was known to be occupied by *N*. *sipedon* prior to the start of the study. We caught snakes on all five days of trapping, and the site was densely populated (estimated 0.22 snakes per m^2^ in 2015, see [18] for more details). Five out of six water samples from this site tested positive for *Nerodia* eDNA. At each of the other 60 sites, all three eDNA samples tested negative for *Nerodia* eDNA (Table 4). We did not detect *N*. *sipedon* using eDNA at one site that was known to be occupied in 2011 but dried completely in the summers of 2013 and 2014.

**Table 4:** Detection results from eDNA and trapping surveys for *N*. *fasciata* and *N*. *sipedon*.

Species	Site	eDNA		Trapping	
		Positive	Negative	Positive	Negative
*Nerodia sipedon*	All	1	60	1	8
	WOHS	5	1	5	0
*Nerodia fasciata*	All	1	29	3	7
	CORC	3	6	1	4
	WCPD	0	9	5	0
	WCGD	0	9	1	4

For the rows with “All” sites, the values are the number of sites with positive and negative detections, where a single positive eDNA sample or single capture of a *Nerodia* counted as a positive. For the rows with individual sites (WOHS, CORC, WCPD, WCGD), the values are the number of eDNA samples or trapping surveys in which *Nerodia* were detected or not.

Of the 30 sites sampled for *N*. *fasciata* eDNA, *Nerodia* eDNA was detected at just a single site (Fig 2). The only eDNA positive site was known to be occupied by *N*. *fasciata* before the study began, based on earlier trapping we conducted in 2013 (Rose and Todd, unpubl. data). Three out of nine water samples from this site tested positive for *Nerodia* eDNA in our qPCR assay. All nine eDNA samples were negative for *Nerodia* eDNA at a second site where *N*. *fasciata* were found in 2013; neither eDNA nor trapping surveys detected *Nerodia* at this location in 2015. In contrast to the eDNA results, we captured *N*. *fasciata* at three out of ten sites that were trapped. At the sole eDNA-positive site (CORC), a single snake was captured once on the first day of trapping. The presence of *N*. *fasciata* at the other two sites (WCPD and WCGD) was not known prior to this study. A single snake was captured once at WCGD, and at WCPD four snakes were captured a total of six times, with at least one capture per day. All nine water samples tested negative for *Nerodia* eDNA at WCPD and WCGD (Table 4).

There was strong support for an effect of species on the detection probability of eDNA surveys (β_sp-eDNA_ = -1.55, 95% CRI = -2.92 –-0.08), with a posterior probability of 0.98 that *N*. *fasciata* had lower detection probability using eDNA than *N*. *sipedon*. The evidence for an effect of species on trapping detection probability was weaker, but still suggests a lower capture probability for *N*. *fasciata* in traps than *N*. *sipedon* (β_sp-trap_ = -0.92, -2.35–0.47; Pr[β_sp-trap_] < 0 = 0.91).

**Fig 4:**
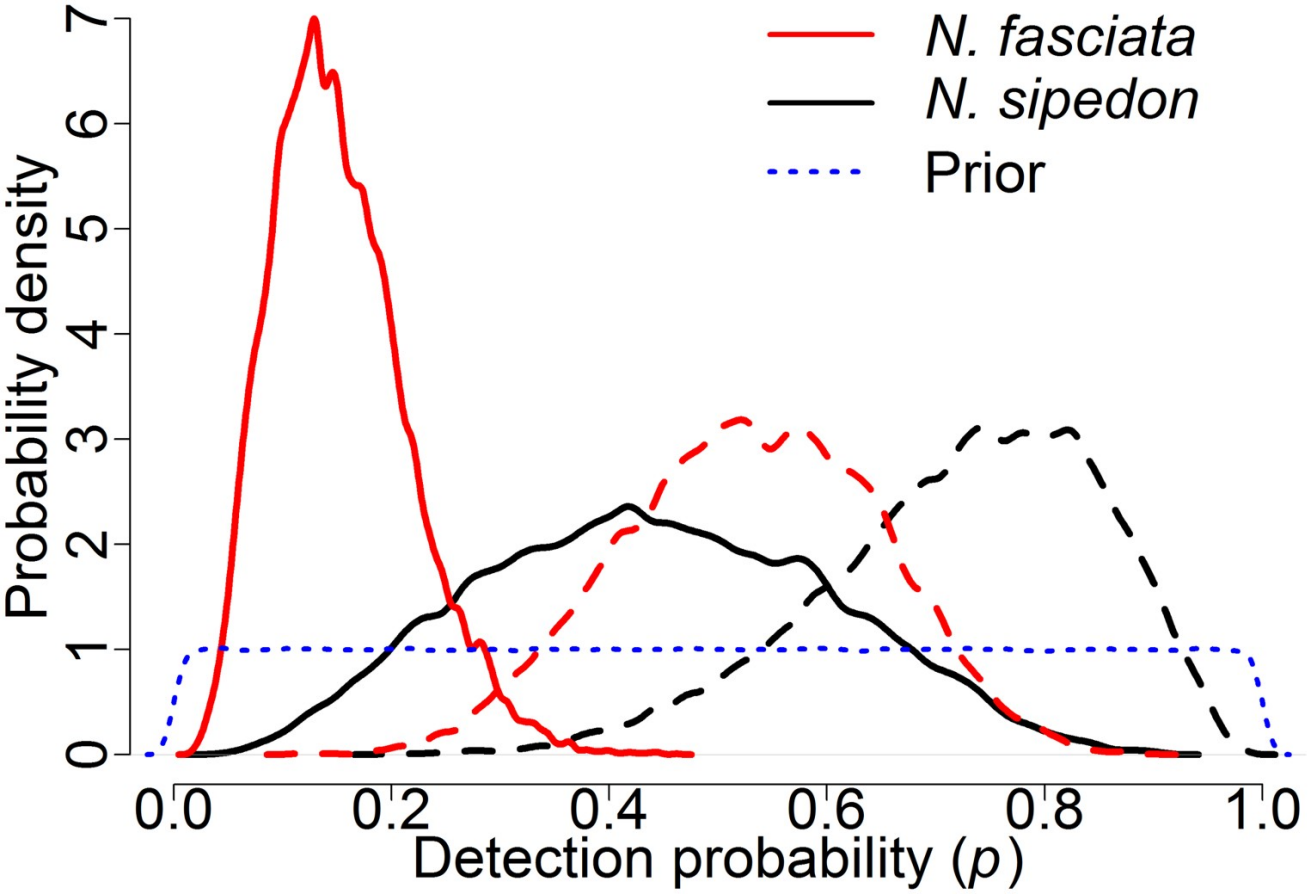
Posterior distributions of detection probability (*p*) for watersnakes. Detection probabilities using eDNA are represented by solid lines and detection probability using trapping are represented using dashed lines. The curves represent the probability of detecting *N*. *fasciata* (red lines) or *N*. *sipedon* (black lines) at a site, given that they are present, for one water sample (eDNA) or one night of trapping with 30 traps. The blue dotted line represents the Uniform(0,1) prior distribution used for detection probability parameters in the Bayesian occupancy model.

Trapping surveys had higher detection probability than eDNA surveys according to the integrated occupancy model, and the difference was greater for *N*. *fasciata* than *N*. *sipedon* (Table 3, Fig 4). The posterior probability that *p*_*trap*_ was greater than *p*_*eDNA*_ was 0.99 (Δ*p*_*fas*_ = 0.38, 0.11–0.63) for *N*. *fasciata* and 0.91 (Δ*p*_*sjp*_ = 0.29, -0.13–0.65) for *N*. *sipedon*. Given that a site is occupied, the median probability of detecting *N*. *sipedon* during a single night of trapping with 30 traps was 0.74 (0.44–0.93). With five nights of trapping at an occupied site, the probability of capturing at least one snake was 0.99 (0.94–0.99). In contrast, the median probability of detecting *N*. *sipedon* eDNA in a single water sample from an occupied site was 0.44 (0.15–0.75). The median probability of detecting *N*. *sipedon* eDNA at least once in three water samples was 0.78 (0.39–0.99), and at least once in nine water samples was 0.99 (0.77–0.99).

For *N*. *fasciata*, the median probability of detecting snakes during a single night of trapping with 30 traps was 0.53 (0.30–0.75). In five nights of trapping a site occupied by *N*. *fasciata*, the median predicted probability of capturing at least one snake was 0.98 (0.83–0.99). The median probability of detecting *N*. *fasciata* eDNA in a single water sample from an occupied site was 0.14 (0.05–0.29), much lower than the probability of detecting this species in a single night of trapping. The median probability of detecting *N*. *fasciata* eDNA at least once in three water samples was 0.37 (0.15–0.65), and at least once in nine water samples was 0.76 (0.39–0.96).

After accounting for the imperfect detection of both sampling methods, the integrated model estimated the occupancy probability for both *Nerodia* species combined to be 0.07 (0.02–0.15). Out of the 91 sites that were surveyed, the model’s median prediction was that 5 sites (4–10) were occupied by *Nerodia* of either species, compared to the four sites where snakes were captured. For *N*. *fasciata*, 4 sites (3–6) out of 30 were predicted to be occupied, while for *N*. *sipedon*, just 1 site (1–5) out of 61 was predicted to be occupied.

Compared to the integrated model, the eDNA-only model, which lacked information about the two sites known to be occupied by *N*. *fasciata* from trapping surveys, overestimated the median detection probability of *N*. *fasciata* (0.40, 0.15–0.70) and *N*. *sipedon* (0.61, 0.30–0.87) from a single eDNA sample at an occupied site and only predicted two sites to be occupied, one for each species (Table 5). In contrast, the model fit to trapping data only predicted similar probabilities of detecting *N*. *fasciata* (0.52, 0.29–0.75) or *N*. *sipedon* (0.73, 0.45–0.94) in a single night of trapping as the integrated model. The occupancy model fit to trapping data from 19 sites predicted a much higher occupancy probability than the integrated or eDNA-only models, which included data from 91 sites (Table 6).

**Table 5:** Parameters, prior distributions, and posterior summaries for detection and occupancy parameters from an occupancy model based on eDNA surveys only.

Parameter	Description	Prior	Median	SD	2.50%	97.50%
*p*_eDNA_	Mean detection probability of eDNA	Uniform(0,1)	0.61	0.15	0.30	0.87
α_eDNA_	Intercept of eDNA linear predictor	--	0.46	0.71	-0.97	1.81
β_fas, eDNA_		Normal(0,1)	-0.88	0.74	-2.34	0.55
ψ	Occupancy probability	Uniform(0,1)	0.03	0.02	0.00	0.08
N_all_	Estimated number of sites occupied by watersnakes	--	2	0.94	2	4
N_fas_	Estimated number of sites occupied by *N*. *fasciata*	--	1	0.57	1	2
N_sip_	Estimated number of sites occupied by *N*. *sipedon*	--	1	0.63	1	2

**Table 6:** Parameters, prior distributions, and posterior summaries for detection and occupancy parameters from an occupancy model based on trapping surveys only.

Parameter	Description	Prior	Median	SD	2.50%	97.50%
*p*_trap_	Mean detection probability of trapping	Uniform(0,1)	0.73	0.13	0.45	0.94
α_trap_	Intercept of trapping linear predictor	--	1.00	0.72	-0.37	2.48
β_fas, trap_	Species effect on *p*_trap_	Normal(0,1)	-0.92	0.73	-2.29	0.56
Ψ	Occupancy probability	Uniform(0,1)	0.24	0.09	0.07	0.42
N_all_	Estimated number of sites occupied by watersnakes	--	4	0.39	4	5
N_fas_	Estimated number of sites occupied by *N*. *fasciata*	--	3	0.32	3	4
N_sip_	Estimated number of sites occupied by *N*. *sipedon*	--	1	0.20	1	1

## Discussion

We have presented a method for integrating multiple survey methods to gain better inference into the occupancy of introduced species. Our results demonstrate that eDNA was less effective than aquatic trapping at detecting introduced populations of the semi-aquatic snakes *N*. *fasciata* and *N*. *sipedon* in California, USA. Estimating detection probabilities for eDNA and trapping was challenging for *N*. *sipedon*, which was only detected at one site out of 61 surveyed. As a result, parameter estimates for *N*. *sipedon* were less precise than those for *N*. *fasciata*, which was detected at three out of 30 sites surveyed. The difference in detectability between methods was greater for *N*. *fasciata*. Nine eDNA samples failed to detect *N*. *fasciata* DNA at each of two sites where trapping surveys confirmed the presence of this species. If we had relied solely on eDNA to determine which sites were occupied by *N*. *fasciata*, we would have estimated an inflated detection probability for eDNA, and erroneously concluded that their distribution was limited to few small ponds, rather than a stretch of a stream drainage extending more than 4 km. Falsely concluding that an introduced species is absent from a site could lead to greater expenses for control efforts or a failure to eradicate a species before it becomes invasive [33]. Thus, combining eDNA with another, independent survey method might be more prudent when surveying for introduced species [34]

Given the potential for false negatives, replication should be a key component of any eDNA study design. Replicate eDNA samples from the same site should always be collected, and detection/non-detection data should be analyzed in an occupancy framework to allow estimating detection probability. Our study adds to a growing number that highlight the importance of accounting for false negatives when using eDNA to document species presence [7,35]. Ideally, spatial and temporal replicates would be collected as well, to quantify how much detection can vary within an occupied site. The length of time eDNA persists in the environment is variable [36,37], as is the distance at which eDNA is detectable downstream from a source in lotic habitats [38,39]. In a hierarchical occupancy model, one can estimate the heterogeneity in detection attributable to spatial and temporal variation within a site [28]. This would allow researchers to scale the number of replicate samples to the size of the body of water, and to determine whether a single site visit is sufficient to detect the focal species if it is present.

Both survey methods had a higher probability of detecting *N*. *sipedon* than *N*. *fasciata*. The higher probability of detecting *N*. *sipedon* through both trapping and eDNA could be a function of the very high density of *N*. *sipedon* in the single known locality occupied during this study. Central California experienced an exceptional drought from 2012–2015 [17], and during this period, the wetland occupied by *N*. *sipedon* shrank substantially, increasing capture rates of this species [19]. In contrast to *N*. *sipedon*, the wetlands occupied by *N*. *fasciata* did not appear to decrease in area during the drought; likely because they receive greater inputs of water from the surrounding suburban developments. Thus, the lower detectability of *N*. *fasciata* might be a result of larger wetlands with lower density watersnake populations. An alternative explanation for the lower detectability of *N*. *fasciata* eDNA is that although our qPCR assay amplified *N*. *fasciata* DNA from tissue samples, it was less sensitive to eDNA from this species than it was for *N*. *sipedon*. Unfortunately, we did not obtain *N*. *fasciata* to create lab-positive water samples during our study. In the future, it would be valuable to test lab-positive water samples for *N*. *fasciata* eDNA, and it may be beneficial to develop distinct target sequences and primers for each species. This would not explain the lower detection probability of *N*. *fasciata* using aquatic traps however.

Although we failed to detect watersnakes in two occupied sites, further testing of our qPCR assay might improve its sensitivity. For example, it was only after initially focusing on avoiding false-positive results (using RNase-H dependent qPCR) that we discovered a conventional PCR, pre-amplification step was necessary to detect *Nerodia* eDNA in lab-positive controls. We recommend that extensive lab testing precede broad deployment of eDNA sampling in the field. Our ultimate goal was to estimate the distribution of two introduced species for which we have little data beyond incidental observations reported to state and federal wildlife agencies. Because data on the distribution of these introduced species is vital for preventing further spread and establishment, we were eager to sample as wide an area as possible. We were also encouraged by recently published studies demonstrating the effectiveness of eDNA for rare and secretive species (e.g., [4,40]). In retrospect, a more fruitful first step would have been to focus on lab or mesocosm experiments where the density of snakes in a fixed volume of water is controlled. This could be followed by collecting many replicate water samples from a smaller number of sites, some of which were known to be occupied by water snakes. This would have allowed us to estimate the detection probability of our eDNA assay in the field and calculate the number of replicate water samples to collect from each site to reach a threshold of certainty that a site is unoccupied, given non-detection in all samples. The undoubted success of eDNA for detecting many species in many systems does not obviate the need for careful testing in a new study system.

The labor cost for eDNA sampling relative to traditional survey methods might also be higher than expected. While field collection of eDNA was faster and allowed covering a broader area than more field-labor intensive trapping in this study, the amount of time spent in the lab processing water samples was considerable. We did not carefully track the number of hours spent on each activity, but filtering hundreds of water samples, extracting DNA from filters, and preparing PCR plates required several hundred person-hours of effort. A more careful accounting of the costs, both in equipment/supplies and time, of each method would be valuable for researchers designing a study that maximizes the information gained under a fixed budget. High-throughput sequencing methods can provide community-level information from eDNA samples and might be a more cost-effective method if multiple species are of interest [41,42].

One limitation of our study is that we did not sequence the PCR product from positive samples to confirm that our assay detected the target *Nerodia* DNA sequence and not another DNA sequence from a non-target organism. We acknowledge that sequencing the PCR product from a positive assay would be a valuable final step for confirming the presence of *Nerodia* DNA. However there were multiple lines of evidence that our assay did detect *Nerodia* and not closely related *Thamnophis* species, which had the most similar sequence. Our PCR assay run on tissue samples only amplified *Nerodia* DNA. Lab-control water samples from aquaria containing *Nerodia sipedon* all returned positive results while those from aquaria containing *Thamnophis sirtalis* or *T*. *elegans* were negative. Finally, positive field samples only came from sites known to be occupied by both *N*. *fasciata* and *N*. *sipedon* based on trapping surveys. We are therefore confident that our results do not represent false positives, which can present a serious issue for occupancy models [16]. In contrast, our study clearly shows that false negatives are a serious concern for eDNA sampling for *Nerodia*, given that all eDNA samples came up as negative at two sites known to be occupied by *N*. *fasciata* based on trapping surveys.

Environmental DNA has proven to be effective for detecting many aquatic species [1,2,5], but the efficacy of using eDNA to detect semi-aquatic snakes has been mixed. Environmental DNA assays of water samples had a high probability of detecting the presence of invasive Burmese pythons (*Python bivittatus*) in southern Florida, USA [8,43]. In contrast, eDNA methods failed to detect the presence of threatened giant gartersnakes (*Thamnophis gigas*) in the Sacramento Valley, California, USA, [44]. The latter study collected water samples from similar aquatic habitats to those sampled in the current study. Despite some false negatives, we believe that eDNA has promise for detecting semi-aquatic reptiles such as *Nerodia*. Still, researchers should not abandon established, albeit labor-intensive, methods without first establishing that eDNA has similar efficacy for detecting their species of interest. We recommend that those interested in pursuing eDNA sampling begin with a small-scale pilot study that allows for quantifying the detection probability of eDNA samples under a range of real-world conditions. We have shown that combining eDNA surveys with other methods has the potential to not only quantify the effectiveness of eDNA, but to also provide further insight into both the detection process and the distribution of rare or introduced species than can any single method alone. Ultimately, it is not the novelty or age of the method that matters, but the inference we can gain from the data we collect. Conserving biodiversity demands accurate, timely data on the distribution of native and invasive species, and we need to use all of the tools available to us in pursuit of this goal.

## Supporting information

S1 File Data and R code to replicate occupancy analysis of eDNA and trapping data. Zipped folder includes three JAGS model files, one R file containing trapping and eDNA survey data, one R file with code to run all analyses, and a metadata file explaining the contents of each and how to repeat analyses.
(ZIP)
